# Can Exercising and Eating Healthy Be Fun and Indulgent Instead of Boring and Depriving? Targeting Mindsets About the Process of Engaging in Healthy Behaviors

**DOI:** 10.3389/fpsyg.2021.745950

**Published:** 2021-10-05

**Authors:** Danielle Z. Boles, Maysa DeSousa, Bradley P. Turnwald, Rina I. Horii, Taylor Duarte, Octavia H. Zahrt, Hazel R. Markus, Alia J. Crum

**Affiliations:** ^1^Department of Psychology, Stanford University, Stanford, CA, United States; ^2^Department of Psychology, Springfield College, Springfield, MA, United States; ^3^Department of Psychology, University of Minnesota Twin Cities, St. Paul, MN, United States; ^4^Department of Organizational Behavior, Stanford Graduate School of Business, Stanford, CA, United States

**Keywords:** health, intervention, eating behavior, physical activity, mindset

## Abstract

This paper investigates mindsets about the process of health behaviors—the extent to which people associate physical activity and healthy eating with appealing (pleasurable, fun, indulgent) versus unappealing (unpleasant, boring, depriving) qualities—to promote greater engagement. Study 1 (*N* = 536) examined how mindsets about physical activity and healthy eating relate to current and future health behavior. Study 2 (*N* = 149) intervened in actual fitness classes to compare the effects of brief appeal-focused and health-focused interventions on mindsets about physical activity and class engagement. Study 3 (*N* = 140) designed nutrition education classes that emphasized either the appeal or the importance of fruits and vegetables for health and compared its effects on mindsets about healthy eating and actual fruit and vegetable consumption. Holding more appealing mindsets about health behaviors predicts subsequent physical activity and healthy eating (Study 1). An intervention targeting mindsets about the appeal of physical activity promotes greater participation in fitness classes than emphasizing the importance of meeting activity guidelines (Study 2). Meanwhile, interventions targeting mindsets about the appeal of healthy eating increases in-class fruit and vegetable selection more than emphasizing the importance of eating nutritious foods (Study 3), however additional work is needed to sustain such changes in eating behavior. These studies suggest mindsets about the process of health behaviors can be influential and changeable factors in motivating physical activity and healthy eating.

## Introduction

Messages reminding us of the importance of engaging in health behaviors are ubiquitous throughout the lifespan. From health education for youth to fitness classes for adults, people are bombarded with public health messages touting the health benefits of physical activity and healthy diets (e.g., CDC.gov, ChooseMyPlate.gov). Underlying these well-intentioned messages is the assumption that if people deem health as important, are aware of exercise and nutrition guidelines, and have access to healthy options, then they will make healthier choices. Yet the sobering truth is that despite considerable effort and resources devoted to educating people about the importance of health behaviors, physical inactivity and unhealthy eating continue to represent major public health challenges. The vast majority (nearly 80%) of Americans, for instance, still do not meet physical activity guidelines or eat enough fruits and vegetables (Moore and Thompson, 2015). This paper proposes that an overlooked and under-targeted driver of healthy behaviors such as diet and exercise are people’s mindsets about the process of engaging in those behaviors.

One’s experience of the world is, in part, a product of the way one construes that world (Markus and Zajonc, 1985; Jamieson et al., 2018; Dweck and Yeager, 2019). Mindsets are a specific type of construal or belief, defined here as one’s core assumptions about the nature of things or categories that activate a specific set of attributions, expectations, and goals (Murphy and Dweck, 2010; Crum et al., 2013). The most widely studied mindsets in the field have, to date, involved core assumptions about the nature of human qualities or characteristics, as in “growth” versus “fixed” mindsets of intelligence (Dweck, 2008). A growing body of research shows that people also hold mindsets about a variety of domains and processes beyond intelligence, including mindsets about stress (as enhancing or debilitating) (Crum et al., 2013), aging (as a positive or negative trajectory) (Levy, 2009), and body weight (as within or beyond one’s control) (Auster-Gussman and Rothman, 2018). Here mindsets about the process of health behaviors are defined as the extent to which people view physical activity and eating healthy as relatively *unappealing* (e.g., boring, depriving, and isolating) or relatively *appealing* (e.g., fun, indulgent, and social) processes.

Contrary to what might be assumed, individuals’ mindsets about health behaviors such as diet or physical activity are neither innate nor bound to the objective qualities of a food or activity. Rather, they are socially and culturally informed and reinforced (Markus and Kitayama, 2010). In daily experiences from childhood through adulthood, Americans grow up exposed to cultural products (e.g., TV/movies, social media, official guidelines) and social interactions (e.g., doctors, parents, and teachers) that frequently portray physical activity (Hall, 2013) and eating healthy (O’Dea, 2003; Turnwald et al., 2017) in terms of their health benefits rather than their immediately appealing qualities. In contrast, French culture does not send as strong health-focused messages (Rozin et al., 1999), instead providing children with opportunities for “l’éducation du goût”—an education of taste and appreciation for a wide variety of foods (Puisais, 1995) which leads them to associate healthy foods more with pleasure than with health (Rozin et al., 1999). In Japan, healthy food is highly valued and widely appealing because it connects people and helps them foster a close family atmosphere (Levine et al., 2016). While many people may dread consuming a vegetable dish or begrudgingly drag their feet to a fitness class, some look forward to savoring that same dish or attending the same class. In these cases, the activity or the food is consistent, yet one’s core assumption about it differs. As these insights reveal, changes in mindsets about health behaviors are possible without requiring objective changes to the target behavior (e.g., exercise duration, food ingredients), but instead, by placing emphasis on the appealing qualities of physical activity and eating healthy.

Regardless of how they form, the mindsets people hold matter. Mindsets can influence health by altering attention, motivation, behavior, and physiology in ways that tend to affirm or reinforce the mindset (Crum et al., 2013, 2017). For example, individuals who have a negative mindset about aging may expect their health to worsen no matter what they do, and engage in less health behaviors such as eating well and exercising (Levy and Myers, 2004). Likewise, holding a mindset that stress is debilitating hampers work performance and cognitive functioning under stress (Crum et al., 2013). In the case of mindsets about health behaviors proposed here, we recognize that some mindsets may be more or less helpful for health behavior and intentions. Related health behavior theories such as the Theory of Planned Behavior (TPB), for instance, have found perceived enjoyment of a behavior (also referred to as affective attitude) to be among the most effective subcomponents of TPB in predicting health behaviors (Godin and Kok, 1996), with affective attitudes more strongly predictive of future behavior than instrumental attitudes regarding its health benefits. To expand this research, we identify and intervene on a broad yet concise set of appealing qualities that collectively reveal people’s mindsets about the experience of physical activity and healthy eating. We expect that encouraging the mindset that health behaviors are appealing across a range of qualities (e.g., fun, indulgent, and relaxing) will have a powerful effect on motivation in part because it increases the perceived value and rewards of healthy choices (Berkman et al., 2017), ultimately capitalizing on (rather than constraining) our drive for immediate pleasure and rewards (Custers and Aarts, 2005). If unhealthy behaviors occur in part because of a conflict of impulses (Duckworth and Gross, 2020), then drawing attention to the rewarding aspects of physical activity or healthy eating can help reduce conflicts between valued goals (e.g., being physically fit) and immediate temptations (e.g., an enjoyable experience) (Woolley and Fishbach, 2016, 2018).

However, despite the rich literatures on the benefits of self-determined (Ryan and Deci, 2000), immediately rewarding activities (Berridge and Robinson, 2003), and the limitations of restriction-based approaches (Mann et al., 2015), the promotion of health behaviors continues to hinge on presenting distant or abstract rewards that cannot necessarily be guaranteed or determined, particularly in health education contexts. For instance, the rewards typically used in nutrition and physical education classes are often distant (e.g., heart benefits that aren’t evident until older age), abstract (e.g., what is optimal health supposed to feel like?), and indeterminable (e.g., you can’t know how many diseases you successfully avoided). Nevertheless, health education remains ubiquitous in the United States and is required in over 90% of schools (Wyche et al., 1997). Whereas others have suggested interventions outside of educational contexts (e.g., families, household), here we wonder how the innumerable resources devoted to health education can better attune to people’s mindsets about health behaviors and the effects of prioritizing teaching the pleasures rather than the health benefits of physical activity and nutritious foods.

The present research hypothesizes that: (1) mindsets about the process of health behaviors (or process mindsets) can help explain individual differences in exercise and healthy eating behavior, (2) such mindsets can be changed by altering health messaging to focus on the appeal of health behaviors, and (3) changes toward more appealing mindsets will lead to increased engagement in healthy behaviors. To test these assertions, scales were first validated to measure mindsets about the process of exercising (MPH-Physical Activity) and eating a healthy diet (MPH-Healthy Eating) before conducting two interventions targeting these mindsets. Study 1 examined the extent to which MPH-Physical Activity and MPH-Healthy Eating relate to current and future exercise and diet 3 months later. Studies 2 and 3 were conducted in actual health education settings to examine whether interventions focused on presenting health behaviors as appealing can change mindsets about the process of health and whether targeting mindsets is more effective in motivating health behaviors than instilling the importance of such behaviors for health.

## Study 1: Do Mindsets About the Process of Health Behaviors Predict Physical Activity and Healthy Eating?

Do mindsets about physical activity and healthy eating predict individual differences in health behaviors? And do they do so over and above measures of other well-known predictors of health behavior such as perceived importance of health and self-efficacy (Luszczynska et al., 2005)? Study 1 aimed to test these questions.

### Materials and Methods

#### Participants

Online participants were recruited using Dynata, which invited participants to a research study on health beliefs and healthy lifestyles. The final sample (*N* = 536) included a demographically-balanced sample consisting of adult men and women (aged 25–86 years) of high and low SES across 4 ethnic groups (see Supplementary Material for demographic details). Participants were surveyed again 3 months after the initial survey date, achieving a 53% retention rate (*N* = 285). Demographic information was collected at the beginning of each survey and screened for in the initial survey. To ensure quality of responses, each survey included two attention checks, and participants who failed at least one attention check at baseline or follow-up were excluded from the data prior to analysis. This study was approved by the Institutional Review Board (IRB; #32948) at Stanford University.

#### Procedure

Surveys were administered via Qualtrics, which online participants completed on personal laptops or tablets. After providing consent and indicating their demographic information, participants answered questions about their mindsets about physical activity and healthy eating, as well as their level of engagement in these health behaviors.

#### Measures

*Mindsets about the process of physical activity and healthy eating* were measured at baseline and 3 months later using MPH-Physical Activity and MPH-Healthy Eating scales (Table 1). See Supplementary Material for preliminary studies on item generation, testing, and validation. *Importance of health* was measured at both timepoints *via* a single item asking participants “How important to you is your health?” (1 = *Not at all*, 5 = *Very)*. *Self-efficacy* was measured at both timepoints using the General Self-Efficacy Scale (Jerusalem and Schwarzer, 2014). Example items include “It is easy for me to stick to my aims and accomplish my goals” (1 = *Not at all true*, 5 = *Exactly true*). *Engagement in physical activity* was measured at both timepoints using the International Physical Activity Questionnaire–Short Form (Lee et al., 2011). Respondents reported their average minutes per week of moderate and of vigorous physical activity over the past 30 days. Metabolic equivalence of aerobic physical activity was calculated using average MET values (multiples of resting metabolic rate), which weights each type of physical activity by energy expenditure (Ainsworth et al., 2000). Moderate aerobic activity was set to a MET value of 4.0, while vigorous exercise was set to 8.0 METs per minute (Craig et al., 2003). *Frequency of healthy eating* was measured at both timepoints by asking participants how often they ate a list of 9 categories of healthy foods (low-calorie, low-carbohydrate, low-fat, low-sodium, low-sugar, whole grain, natural/unprocessed, nutritionally-balanced foods, and fruits and vegetables) in the past 30 days (1 = “Never,” 2 = “Rarely,” 3 = “Sometimes,” 4 = “Often,” or 5 = “All of the time”). Items for this scale were developed based on the most common ways healthy foods were described (Turnwald et al., 2017). To reflect a comprehensive range of healthy food categories, we included foods that were healthy because they were chockful of nutrition (e.g., fresh produce), as well as foods considered healthy because they lacked unhealthy ingredients (e.g., low-fat). A mean score was calculated across the 9 categories of healthy foods, with higher means representing more frequent healthy eating behavior.

**TABLE 1:** Items and instructions for mindsets about the process of health scales (MPH-Physical Activity and MPH-Healthy Eating).

**MPH-Physical Activity (MPH-Healthy Eating)**			
**The following statements are different opinions about what it is like to perform physical activity (eat healthy). Please select the option on each row that best describes how you feel about engaging in physical activity (eating healthy). Exercising (Eating healthy) is:**			
**(1)**	**(2)**	**(3)**	**(4)**

1. Very difficult	Somewhat difficult	Somewhat easy	Very easy
2. Very unpleasant	Somewhat unpleasant	Somewhat pleasurable	Very pleasurable
3. Very stressful	Somewhat stressful	Somewhat relaxing	Very relaxing
4. Very depriving	Somewhat depriving	Somewhat indulgent	Very indulgent
5. Very boring	Somewhat boring	Somewhat fun	Very fun
6. Very lonely	Somewhat lonely	Somewhat social	Very social
7. Very inconvenient	Somewhat inconvenient	Somewhat convenient	Very convenient
(8. Very bad tasting	Somewhat bad tasting	Somewhat good tasting	Very good tasting)

*Items are shown for both measures (measured separately). Study 3 adds the item, “bad tasting/good tasting” to MPH-Healthy Eating. Scores are calculated by taking the mean of individuals’ responses to items 1–7 for MPH-Physical Activity and 1–8 for MPH-Healthy Eating.*

#### Statistical Analysis

All analyses were run in R, including analyses of baseline differences, descriptive analyses, correlations, and regression analyses. Pearson correlations with Holm-Bonferroni correction assessed the relationship between mindset and current and future health behavior variables. To test whether process mindsets predict health behavior over and above measures of importance of health and self-efficacy, separate hierarchical linear regressions predicted total aerobic activity (METs/week) and frequency of healthy eating at 3 months (Time 2). Step 1 of both models included importance of health and self-efficacy at Time 1. Step 2 added Time 1 ratings for either MPH-Physical Activity to predict exercise or MPH-Healthy Eating to predict eating behavior.

### Results

Means, standard deviations, and correlations between all variables at two timepoints are presented in Table 2. The mindset measures had a slight to moderate positive correlation with health behavior such that mindsets of appeal were associated with more physical activity and healthier eating. Aerobic activity was most strongly correlated with MPH-Physical Activity and healthy eating behavior was most strongly correlated with MPH-Healthy Eating.

**TABLE 2:** Correlations of mindsets about the process of health scales (MPH-Physical Activity and MPH-Healthy Eating) with health importance, self-efficacy, and health behaviors at T1 and T2.

**Measure**	**N**	**Mean (SD)**	**1**	**2**	**3**	**4**	**5**	**6**	**7**	**8**	**9**	**10**	**11**
**Time 1**													
*Mindsets about the process of health*													
1. MPH-Physical Activity	536	2.62 (0.65)	—										
2. MPH-Healthy Eating	536	2.58 (0.61)	0.64^***^	—									
*Perceived importance and efficacy*													
3. Importance of health	536	3.68 (0.55)	0.22^***^	0.22^***^	—								
4. Self-efficacy	536	3.15 (0.53)	0.25^***^	0.27^***^	0.19^***^	—							
*Health behaviors*													
5. Aerobic Activity (METs)	536	325.00 (515.67)	0.24^***^	0.13^*^	0.10	0.09	—						
6. Frequency of healthy eating	536	3.07 (0.77)	0.33^***^	0.38^***^	0.27^***^	0.34^***^	0.18^***^	—					
**Time 2**													
*Mindsets about the process of health*													
7. MPH-Physical Activity	285	2.58 (0.51)	0.76^***^	0.53^***^	0.24^***^	0.26^***^	0.27^***^	0.30^***^	—				
8. MPH-Healthy Eating	285	2.54 (0.47)	0.54^***^	0.76^***^	0.24^***^	0.33^***^	0.20^**^	0.38^***^	0.61^***^	—			
*Perceived importance and efficacy*													
9. Importance of health	285	3.66 (0.60)	0.24^***^	0.21^**^	0.54^***^	0.28^***^	0.13	0.31^***^	0.21^**^	0.24^***^	—		
10. Self-efficacy	285	31.22 (5.75)	0.31^***^	0.36^***^	0.22^**^	0.71^***^	0.09	0.28^***^	0.30^***^	0.39^***^	0.35^***^	—	
*Health behaviors*													
11. Aerobic activity (METs)	285	427.04 (647.12)	0.28^***^	0.12	0.09	0.07	0.25^**^	0.20^***^	0.24^***^	0.14	0.1	0.13	—
12. Frequency of healthy eating	285	3.00 (0.78)	0.37^***^	0.38^***^	0.29^***^	0.30^***^	0.25^***^	0.76^***^	0.38^***^	0.47^***^	0.32^***^	0.32^***^	0.15^*^

*p < 0.05, **p < 0.01, and ***p < 0.001.

Next, results from hierarchical linear regressions showed that MPH-Physical Activity significantly predicted physical activity behavior (measured by METS) (ΔR^2^ = 0.07, *p* < 0.001) while MPH-Healthy Eating significantly predicted healthy eating (ΔR^2^ = 0.07, *p* < 0.001) 3 months later controlling for measures of self-efficacy and importance of health (Table 3). These results also showed that mindsets about physical activity also significantly predicted future healthy eating behavior, though mindsets about healthy eating were still stronger predictors of the frequency of eating healthy foods.

**TABLE 3:** Hierarchical linear regressions with mindsets about the process of health scales (MPH-Physical Activity and MPH-Healthy Eating) predicting health behavior 3-months later controlling for health importance and self-efficacy.

	**Total aerobic activity (METs/week)**			**Frequency of healthy eating**		
	**β**	***R* ^2^**	**ΔR^2^**	**β**	***R* ^2^**	**ΔR^2^**
**MPH-Physical Activity**						
Step 1		0.00	0.00		0.14	0.14^***^
Importance of health	0.07			0.24		
Self-efficacy	0.05			0.25		
Step 2		0.07	0.07^***^		0.21	0.06^***^
Importance of health	0.01			0.18		
Self-efficacy	−0.01			0.19		
MPH-Physical Activity	0.28			0.27		
**MPH-Healthy Eating**						
Step 1		0.00	0.00		0.14	0.14^***^
Importance of health	0.07			0.24		
Self-efficacy	0.05			0.25		
Step 2		0.01	0.01		0.21	0.07^***^
Importance of health	0.06			0.19		
Self-efficacy	0.03			0.18		
MPH-Healthy Eating	0.10			0.28		
*N*	285			285		

^†^<*0.10*, *p < 0.05, **p < 0.01, and ***p < 0.001.

In terms of attrition, those who completed the second survey did not differ by race [χ^2^(1) = 1.58, *p* = 0.664], in baseline mindsets about physical activity [*b* = 0.04, 95% CI(−0.08, 0.15), and *p* = 0.531] and healthy eating [*b* = −0.01, 95% CI(−0.11, 0.10), and *p* = 0.863], or total aerobic activity [*b* = −15.43, 95% CI(−103.18, 72.33), and *p* = 0.730] and frequency of healthy eating [*b* = −0.09, 95% CI(−0.22, 0.04), and *p* = 0.160] compared to non-respondents. Results indicate a trending difference by gender [χ^2^(1) = 3.02, *p* = 0.082] between respondents and non-respondents, though this did not reach significance.

### Discussion

Results from Study 1 suggest that mindsets about the process of health behaviors relate to current and future behavior and have distinct influence beyond self-efficacy and importance of health. However, behaviors were self-reported and in the case of eating behavior, were assessed using unstandardized scales, which limits our understanding of how mindsets relate to actual behavior.

## Study 2: Leveraging Process Mindsets to Motivate Physical Activity

Study 1 demonstrated that mindsets about the process of performing physical activity and eating healthy relate to self-reported physical activity and eating behavior beyond other behavior change constructs typically harnessed in health education (e.g., perceived importance of health, self-efficacy). Next, Studies 2 and 3 sought to determine whether mindsets can be changed in real-world settings and whether these changes would lead to changes in actual health behavior. In efforts to inspire people to create and stick to their fitness goals, fitness classes often emphasize the benefits and importance of physical activity along with the hard work required to achieve those benefits (e.g., “No pain, no gain”). Nevertheless, no-show rates in fitness centers are notoriously high (DellaVigna and Malmendier, 2006). This study compared this traditional health-focused approach to a novel intervention designed to help evoke the mindset that physical activity is appealing (e.g., fun, social, and pleasurable).

### Materials and Methods

#### Participants and Design

Graduate and undergraduate students (*N* = 149) (36.2% Asian; 32.2% White; 11.4% Hispanic, 4.7% Black, 14.1% Multiracial, 1.3% unknown race) were recruited from 12 university fitness classes (two indoor cycling classes, four tennis classes, and six swimming classes). Half of the classes were randomly assigned to the *appeal-focused* condition (*N* = 71) and half to the *health-focused* condition (*N* = 78). Type of class (cycling, tennis, or swimming) was counterbalanced by condition, and all classes were matched for weekly time commitment (50-min sessions, bi-weekly), attendance policy, and instructor. The final sample reflects the number of enrolled students who were present on the day of the intervention.

#### Procedure

**FIGURE 1:**
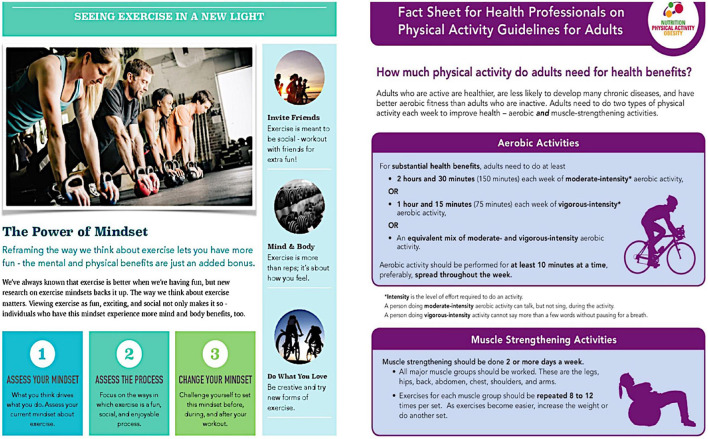
Printed brochures for appeal-focused (left) and health-focused conditions (right).

During the second week of class, attendees were asked if they would participate in a study aimed at understanding ways to improve the University’s physical education program. Participants completed an alteration of consent and could withdraw from the study at any time. Consenting participants completed a short baseline questionnaire before all participants received a 10-min speech and print brochure modified according to condition (Figure 1). Seven weeks later, on the last day of each class, the experimenter collected follow-up measures and debriefed participants. Throughout the 7 weeks, instructors were asked to conduct all classes as they normally would and were not involved in the dissemination of materials or in reinforcing intervention content. Study protocols were approved by the Stanford University IRB (#37035).

#### Intervention Content

Participants in the *appeal-focused condition* received a brief oral presentation and a handout highlighting exercise as a source of pleasure, fun, and relaxation and encouraged them to adopt the mindset that physical activity is appealing (see Figure 1). Presentations were scripted and practiced ensuring consistency in delivery in all classes. Quotes from the handout included suggestions that embodied the core components of the process mindset measure. The presentation reinforced the appealing qualities of physical activity by having participants share with the class what they enjoyed most about exercising.

In the *health-focused condition*, participants received a presentation and handout based on Centers for Disease Control and Prevention’s recommendations for physical activity for adults (at least 150 min of moderate-intensity aerobic exercise or 75 min of vigorous aerobic exercise per week) and listed the health benefits of regular physical activity. The presentation reviewed these guidelines and encouraged the audience to think of additional health benefits of regular physical activity (see Supplementary Material for more details on Study 2).

#### Measures

*Mindsets about the process of physical activity* (MPH-Physical Activity) was measured at two time points—immediately before the intervention was delivered (Week 2 of quarter) and on the last day of class (Week 9 of quarter). Participants who were lost to attrition (*n* = 35) at follow-up were excluded from analyses of change in MPH-Physical Activity. *Class attendance* was taken by the instructors at each fitness class and instructors submitted attendance records for all participants who completed baseline measures. The number of classes attended by each student out of the total amount of classes that occurred post-intervention was converted into a percentage. *Enrollment in future fitness classes* was measured on the last day of class. Participants indicated “*yes*” or “*no*” to whether or not they enrolled in a fitness class next quarter.

#### Statistical Analysis

To calculate participant attrition, we first conducted a mixed effects logistic regression predicting attendance to the follow-up session as a function of condition with the random effect of class. From the attendance data, we were also able to determine differences in attendance rates by condition using a linear mixed effects model with the random effect of class. To test the effects of the intervention on participants’ mindsets, a linear mixed effects model predicted MPH-Physical Activity as a function of time (0 = pre-intervention, 1 = post-intervention) × condition (0 = health-focused, 1 = appeal-focused) with random nested effects of participants within class type (cycling, swimming, or tennis). Effect size (Cohen’s *d*) was computed using Wilson’s effect size calculator (Lipsey and Wilson, 2001). Lastly, we assessed participants’ likelihood of taking a PE class next quarter by running a generalized linear model predicting whether or not participants in each condition planned to take a PE class the following quarter.

### Results

#### Participant Attrition

Participants (*N* = 114) completed the post-measures (67 appeal-focused condition, 47 health-focused condition). Results revealed significantly lower attrition in appeal-focused classes (*N* = 11) than health-focused classes [*N* = 24; OR = 3.12, 95% CI: (1.42, 7.28), *p* = 0.006]. The proportion of participants who failed to complete post-measures did not differ from expected proportions by race [χ^2^(5) = 1.84, *p* = 0.871] nor did participants lost to follow-up significantly differ in their mindset about the process of exercising [*b* = −0.11, 95% CI: (−0.26, 0.04), *p* = 0.164] at baseline than participants who were present for the post-survey. Attendance records show that those who were absent at follow-up on the last day of class missed significantly more (9.5%) classes throughout the quarter [*b* = −9.46, 95% CI(−13.66, −5.25), and *p* < 0.001] than those present at follow-up.

#### Changes in Mindsets About the Process of Physical Activity

**FIGURE 2:**
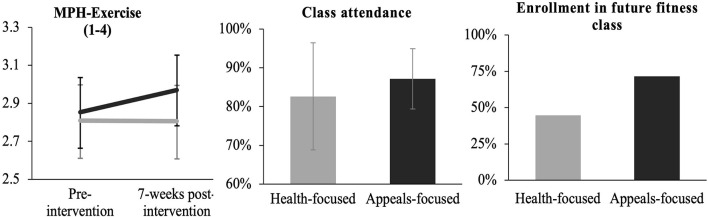
Change in MPH-physical activity, class attendance, and enrollment in future fitness class as a function of condition. Black lines/bars represent the appeal-focused condition, gray lines/bars represent the health focused condition. Error bars represent 95% confidence intervals. Class attendance is the mean percentage of sessions attended out of the total number of sessions. Enrollment in future fitness class is the percentage of participants who indicated “Yes” to taking a class the following quarter.

Results showed that individuals in the appeal-focused class improved their process mindset about exercise to a greater extent [time × condition interaction: *b* = 0.12, 95% CI: (0.02, 0.23), *p* = 0.023, *d* = 0.27] than individuals in the health-focused class (Figure 2). Simple effects tests showed that participants in appeal-focused classes significantly improved their mindset about exercise [*b* = 0.12, 95% CI: (0.05, 0.19), *p* = 0.001], whereas participants in health-focused classes did not change their mindset [*b* = 0.00, 95% CI: (−0.08, 0.07), and *p* = 0.910].

#### Overall Class Attendance

A mixed effects model with the random effect of class type revealed that participants in appeal-focused classes attended a significantly higher percentage of classes than students in health-focused classes [*M*_appeal_ = 87.16%, *SD*_appeal_ = 7.77% versus *M*_health_ = 82.61%, *SD*_health_ = 13.82%; *b* = 4.55%, 95% CI: (0.95, 8.14), *p* = 0.014, *d* = 0.43; Figure 2].

#### Enrollment in Future Fitness Class

A generalized linear mixed effects model predicting self-reported fitness class enrollment next quarter with a random effect of class type revealed that students in appeal-focused classes were more likely to indicate “Yes” when asked “Do you plan on taking a physical education course next quarter,” compared to those in the health-focused condition [71.6% vs 44.7%; *Odds Ratio* = 3.12, 95% CI(1.44, 6.95), *p* = 0.004].

### Discussion

Study 2 demonstrated that a brief, targeted 10-min introduction and handout helped establish more appealing mindsets about physical activity. Seven weeks after the appeal-focused intervention materials were delivered, appeal-focused classes significantly improved participants’ mindsets about the process of engaging in physical activity compared to health-focused fitness class. No improvements were observed among those in the health-focused condition, whose mindsets remained relatively the same 7 weeks post-intervention. Moreover, individuals in appeal-focused classes had greater adherence (class attendance) and approximately three-times greater odds of enrolling in a future fitness class compared to individuals in classes that emphasized the importance of physical activity for good health. Participant attrition rates further support the finding that promoting appealing mindsets about physical activity increases exercise adherence. Nearly three times more participants in the health-focused condition failed to complete follow-up measures, either due to dropping the class or missing attendance on the final class day.

Study 2 had several limitations due in part to constraints of real-world settings. First, participants self-enrolled so randomization to conditions occurred at the level of class rather than the individual. The multilevel model specifies the nesting of participants within class type to account for this design. Second, MPH-Physical Activity was collected only at weeks 2 and 9, so it is unknown whether the intervention prompted immediate and sustained changes in process mindset or whether process mindsets changed gradually over time. Mediation models testing the extent to which changes in attendance were mediated by changes in mindset were not possible given the attrition occurred before the second mindset measure was taken.

## Study 3: Leveraging Process Mindsets to Motivate Healthy Eating

Study 2 presented evidence suggesting that mindsets about physical activity can be changed with accompanying changes in class attendance and enrollment in future fitness classes. Can mindsets about healthy eating be changed? Informed by theory and results on process mindset from Studies 1 and 2, this study designed an intervention to present healthy eating education in a manner that focuses on the appealing aspects of eating healthy foods. Compared to a class that focused on the importance and nutritional value of healthy eating, emphasizing pleasurable, fun, and social experiences with fruits and vegetables was expected to enhance mindsets of appeal about the process of healthy eating as well as inspire healthy food selection. This study also measured process mindsets immediately after the intervention to test if changes in mindset due to the intervention mediated subsequent changes in eating behavior.

### Materials and Methods

#### Participants and Design

This study was conducted in partnership with a youth education program through Stanford University and could accommodate up to 180 students. The final sample size reflects the number of participants who enrolled in the class and were present on the day of the study. Participants consisted of 140 adolescents from middle schools across California between 13–14 years of age (57.9% female; 52.1% Asian American, 24.3% European American, 5.0% African American, 4.3% Latinx American, and 14.3% mixed race or other) who elected to enroll in one of six 45-min classes entitled “Mind, Body, Food, and You.” Participants were enrolled in novel classes randomly assigned to either a health-focused condition or an appeal-focused condition with counterbalancing to control for time effects. A total of 75 participants were randomized into the health-focused condition and 65 were randomized into the appeal-focused condition. Participants did not differ by gender or ethnicity between conditions.

#### Procedure

All participants completed youth assent forms and could opt out of participation in the study at any time. After class, parents/guardians received letter notices that their child had participated in a research study and that they have the right to have their child’s data removed from the study. All classes were scripted and taught by the same two instructors, with both instructors delivering both conditions. While the activities and foods served in class were consistent across all classes, the overarching framing of the intervention content, such as how fruits and vegetables were described, differed according to class condition (health-focused or appeal-focused class). In addition to MPH-Healthy Eating, participants completed measures on self-reported intake of fruits and vegetables immediately before and after the class. Participants were contacted 1 week later to complete a 10-min follow-up survey in exchange for $10. All study protocols were approved by Stanford’s IRB (#37035).

#### Intervention Content

The *appeal-focused condition* was designed to evoke the mindset that healthy eating is appealing. The instructor focused on showcasing the tasty, fun, and social aspects of eating fruits and vegetables. Class began with a discussion of students’ favorite dishes, during which the instructor highlighted the appealing qualities of any mentions of fruits and vegetables. Participants discussed how healthy foods facilitate social collaboration (e.g., sharing recipes, social media posts) and seasonal celebrations (i.e., squashes in the fall, melons in the summer). For each fruit and vegetable presented during the lesson, students’ attention was directed to the food’s rich flavor, color, and texture. In addition, participants learned how a variety of foods grow and that gardening is an opportunity to relax outdoors, express creativity, and connect with others. Following the lesson was an opportunity to sample fruits and vegetables (dragon fruit, frozen grapes, jicama, and beets). Each food was described emphasizing its sensory appeal (i.e., “served cold, jicama sticks are a refreshing snack on a warm, sunny day”) before participants could select which foods to sample. Finally, participants were given a recipe card and shown how to make a fun and delicious “Summer Sipper,” depicting a fruit and vegetable smoothie as a colorful umbrella drink. Participants were offered samples in class and encouraged to make it at home (see Supplementary Material for more information).

The *health-focused condition* was similar in format, but the information provided focused on nutritional quality and health benefits of fruits and vegetables. The lesson detailed MyPlate guidelines, touting each food groups’ nutrient profiles and implications for health and disease prevention. Fruits and vegetables were praised for being low-sugar, low-fat, and low-calorie before reviewing strategies for increasing one’s fruit and vegetable intake (e.g., meal prepping). The instructor also reviewed self-monitoring and portion control strategies (e.g., using smaller plates, designating half for fruits and vegetables). During the food sampling, fruits and vegetables were presented as healthy alternatives to junk foods and described in terms of their nutrient content and health benefits before students could select which foods to sample. Finally, the demonstration of the fruit and vegetable smoothie focused on tracking servings and abiding by proper measurements. The recipe card showed a “Healthy Green Smoothie” with a bowl of spinach and a MyPlate diagram. Participants were then offered samples to try the smoothie in class and encouraged to make it at home (see Supplementary Material for more information).

#### Measures

*Mindsets about the process of eating healthy* were measured using MPH-Healthy Eating. Cronbach’s alphas were .76 pre-intervention and .79 post-intervention. *In-class healthy food consumption* was assessed by asking participants to mark an “X” by each fruit and vegetable they tried (i.e., dragon fruit, grapes, jicama, and beets) then calculating the sum of fruits and vegetables they tried during the in-class food sampling activity. *Self-reported fruit and vegetable consumption* was measured by surveying how many cups of fruits and vegetables they ate in a typical day in the previous week, in accordance with USDA official recommendations from ChooseMyPlate.gov. This was measured at the pre-intervention survey and at 1-week post-intervention. Intentions to eat healthy were measured in the post-intervention survey by asking participants how many cups of fruits and vegetables they intend to eat over the next 7 days. *Recreating healthy recipes* was measured 1 week later, asking participants whether they made the smoothie recipe demonstrated in class and if so, how many times they made it in the following week. This measure supplements self-reported intake of fruits and vegetables and overcomes limitations of self-reported servings stemming from difficulties in identifying and measuring ingredients of all meals consumed throughout the week.

#### Statistical Analysis

To assess the effect of condition on changes in MPH-Healthy Eating, intentions, and eating behavior, we performed linear mixed effects models using the *lme4* package in R (Version 3.4.1). Models predicted outcomes as a function of time (0 = pre-intervention, 1 = post-intervention) × condition (0 = health-focused, 1 = appeal-focused) with the random nested effect of participants within different classes. Chi-square goodness-of-fit tests were performed to determine any differences by condition in response rates to the follow-up survey by comparing the observed proportion of follow-up respondents to the expected proportion for each condition based on in-class attendance. A chi-square goodness-of-fit test was also used to compare the proportion of students from each condition who tried the healthy recipe at home to the expected proportions based on follow-up response rates, while a Poisson regression predicted the frequency of creating the recipe as a function of condition.

Lastly, causal mediational analyses were run using R’s *mediation* package (Version 4.4.7) to determine if the association between class condition (X) and frequency of making the healthy recipe (Y) was mediated by the change in MPH-Healthy Eating from baseline to post-class (M). This package implements a bootstrapping method according to protocol by Preacher and Hayes (2004), which does not require the data to be normally distributed and is particularly suitable for small sample sizes (e.g., *N* = 65 respondents to follow-up survey). The indirect effect was computed using a bootstrap estimation with 5,000 samples and bias-corrected 95% confidence intervals.

### Results

#### Effect of Condition on Mindsets About Eating Healthy

**FIGURE 3:**
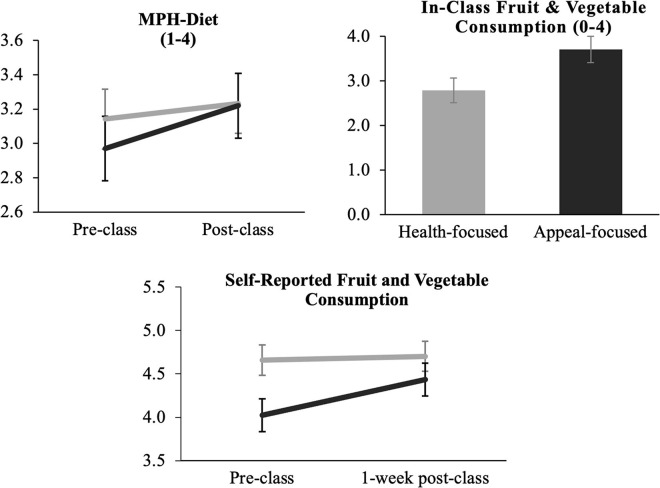
MPH-healthy eating and in-class and self-reported fruit and vegetable consumption as a function of condition. Black lines/bars represent appeal-focused condition, gray lines/bars represent health focused condition. Error bars represent 95% confidence intervals.

A linear mixed effects model showed a significant condition × time interaction [*b* = 0.16, 95% CI (0.06, 0.25), *p* = 0.001, and *d* = 0.39], such that individuals in the appeal-focused class improved their process mindset about healthy eating over time to a greater extent than individuals in the health-focused class (Figure 3). The health-focused condition had higher MPH-Healthy Eating scores at baseline than the appeal-focused condition, though this difference was not statistically significant [*t*(128.64) = 1.66, 95% CI (−0.02, 0.25), *p* = 0.10]. Tests for simple effects by condition show that participants in the appeal-focused condition showed significant increases in MPH-Healthy Eating from pre- to post-class [*b* = 0.25, 95% CI (0.17, 0.32), *p* < 0.001]. The health-focused condition also exhibited significant improvements in MPH-Healthy Eating before and after class [*b* = 0.09, 95% CI (0.03, 0.15), *p* = 0.003], however, improvements were greater in the appeal-focused condition.

#### In-Class Healthy Food Consumption

Three separate generalized linear models predicted fruit, vegetable, or total fruits and vegetables with random effect of participant. Results revealed that participants in appeal-focused classes consumed significantly more fruits [*M* = 1.89, *SD* = 0.40; *b* = 0.41, 95% CI(0.20, 0.62), *p* < 0.001, and *d* = 0.66], vegetables [*M* = 1.80, *SD* = 0.54; *b* = 0.52, 95% CI(0.28, 0.76), *p* < 0.001, and *d* = 0.71], and total combined fruits and vegetables [*M* = 3.72, *SD* = 0.86; *b* = 0.93, 95% CI(0.52, 1.34), *p* < 0.001, and *d* = 0.76] in class compared to those in health-focused classes (fruits: *M* = 1.49, *SD* = 0.78; vegetables: *M* = 1.29 *SD* = 0.83; total healthy foods: *M* = 2.80, *SD* = 1.44) (Figure 3). Learning about the enjoyable aspects of healthy eating corresponded with 39.5% more participants consuming the vegetable samples and 26.8% more participants consuming the fruit samples than those in the traditional health-focused curriculum.

#### Response to Follow-Up Survey

Of the 140 participants, 63 (45.0%) completed the 1-week follow-up survey, 38 (50.7%) from the health-focused class and 25 (38.5%) from the appeal-focused class. Chi-square goodness-of-fit tests showed that responsiveness to the follow-up survey did not differ by condition [χ^2^(1) = 1.14, *p* = 0.285]. Those who were lost to follow-up did not significantly differ from respondents in MPH-Healthy Eating scores at baseline [*t*(122.72) = 0.030, 95% CI (−0.14, 0.14), and *p* = 0.977] or immediately post-class [*t*(128.76) = 0.515, 95% CI (−0.10, 0.18), *p* = 0.607], nor did they differ in baseline [*t*(116.06) = −1.070, 95% CI(−1.08, 0.32), and *p* = 0.286] or intended [*t*(128.85) = 0.239, 95% CI(−0.53, 0.68), and *p* = 0.812] fruit and vegetable consumption.

#### Changes in Self-Reported Healthy Eating Intentions and Behavior

The overall sample reported eating a daily average of 4.23 cups of fruits and vegetables combined prior to the intervention, which is aligned with USDA recommended servings. *T*-tests show no difference in fruit and vegetable consumption by condition at baseline [*t*(96.20) = 1.397, 95% CI(−0.21, 1.22), and *p* = 0.166]. Intentions to eat healthier increased to a greater extent pre- to post-intervention for participants in the appeal-focused class than participants in the health-focused class [condition × time interaction: *b* = 0.61, 95% CI (0.14, 1.07), *p* = 0.012]. Simple effects tests revealed that the health-focused class intended to eat more fruits and vegetables after class compared to baseline [*b* = 0.92, 95% CI (0.60, 1.24), and *p* < 0.001], though the increase in intentions to eat more healthy foods were stronger among the appeal-focused class [*b* = 1.54, 95% CI (1.20, 1.87), and *p* < 0.001]. However, the condition × time effect on self-reported fruit and vegetable consumption between baseline and follow-up was not significant [*b* = 0.37, 95% CI (−0.63, 1.37), and *p* = 0.473] (Figure 3).

#### Likelihood of Recreating Healthy Smoothie Recipe

Among the 63 participants who responded to the follow-up survey, 17 (27%) reported making the fruit and vegetable smoothie, eight from the health-focused condition and nine from the appeal-focused condition. Chi-square tests revealed no differences by condition in how many participants reported making the smoothie at home [χ^2^(1) = 1.25, *p* = 0.265] compared to expected proportions based on follow-up response rates of each condition. Data for the frequency in which participants followed the recipe yielded significant zero-inflation. To account for this, a Poisson regression was used to assess frequency of making the recipe by condition. This model revealed that participants in the appeal-focused class made the smoothie recipe (*M* = 0.56, *SD* = 0.87) marginally more frequently [*b* = 0.76, 95% CI (−0.05, 1.60), and *p* = 0.068] than participants in the health-focused class (*M* = 0.26, *SD* = 0.55), though this effect was not significant.

#### Mindset Mediation Effects

**FIGURE 4:**
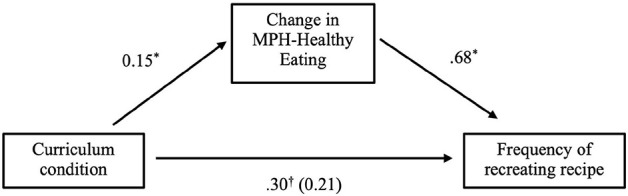
Change in MPH-healthy eating partially mediates effect of condition on the frequency of recreating the healthy smoothie recipe. †*p* < 0.10, *p < 0.05, **p < 0.01, and ***p < 0.001.

A 5,000-sample bootstrapping mediation analysis (Preacher and Hayes, 2004) was used to explore whether changes in mindset could help explain the frequency of recreating the healthy smoothie. This model determined if the association between class condition (X) and frequency of making the healthy recipe (Y) was mediated by changes in MPH-Healthy Eating from baseline to post-class (M). Results revealed that positive changes in MPH-Healthy Eating partially mediated the effect of condition on making the smoothie. The model showed a non-significant but trending indirect effect of class condition on making the smoothie [indirect effect = 0.10, 95% CI (0.00, 0.24), and *p* = 0.051] (Figure 4).

### Discussion

Results from Study 3 suggest that a nutrition education class highlighting the immediately appealing attributes of healthy foods (e.g., taste, variety, and visual appeal, potential for fostering social connection) induced greater changes toward an appeal-focused mindset about the process of eating healthy than did a class on dietary guidelines and nutritional properties of healthy foods. Moreover, focusing on the appealing qualities of nutritious foods prompted students to sample more fruits and vegetables in the class and shows potential for increasing students’ willingness to try nutritious recipes at home. However, there were no differences by condition in self-reported fruit and vegetable intake 1 week later.

Interestingly, those in health-focused classes also demonstrated improvements in mindset, perhaps because they also sampled healthy foods (food sampling activity was held consistent across conditions but is not required in traditional nutrition education). Although changes in mindsets were significantly greater in the appeal-focused condition, results suggest some baseline differences in mindset between conditions. Although not significant, these differences could suggest issues with randomization. Therefore, we cannot rule out regression to the mean as a potential explanation for mindset effects. Lastly, this study was limited by the high attrition of participants between the intervention and 1-week follow-up, and thus follow-up effects should be interpreted while acknowledging this limitation.

## General Discussion

Mindsets about the process of health behaviors have meaningful implications for individuals’ engagement in exercise and eating behaviors. Study 1 provided initial evidence for the relationship between process mindsets and health behaviors like physical activity and healthy eating. Holding the mindset that physical activity and/or healthy eating is a relatively more appealing process (e.g., relaxing, indulgent) predicted greater engagement in those health behaviors up to 3 months later over and above measures of the perceived importance of health and self-efficacy. Studies 2 and 3 moved beyond correlational inference to demonstrate that process mindsets can be shaped in physical and nutrition education settings and that such changes can inspire health behaviors in community samples. In Study 2, a brief intervention at the beginning of class highlighting the enjoyable aspects of physical activity (as opposed to the requirements and benefits of exercise) improved mindsets about the process of exercising, attendance rates, and increased enrollment in future fitness classes. In Study 3, a nutrition education class highlighting the enjoyable, exciting, and tasty attributes of healthy foods (as opposed to the requirements and benefits of eating a balanced diet) improved mindsets about the process of eating healthy, led to increases in healthy food selection during class, and marginally increased consumption of a healthy smoothie following class, although changes in self-reported fruit and vegetable consumption were not significant. Taken together, these studies provide some initial support for the assertions that mindsets about the process of exercising and eating healthy (1) can be measured concisely and reliably, (2) meaningfully relate to health behavior, and (3) can be changed to improve exercise adherence and, to some degree, healthy food choice.

### Implications for the Science and Practice of Health Behavior Change

This work has important implications for the science and practice of behavior change. Construing health behaviors as something to take pleasure in rather than something we dread doing aligns with research on the value of immediate rewards in shaping behavior (Woolley and Fishbach, 2016) and thus may seem obvious in retrospect. However, the dominant strategies and discourse aimed at motivating healthy behaviors not only ignores the wealth of appeal underlying these behaviors, they sometimes undercut it (Turnwald et al., 2017). Mindset theory (Murphy and Dweck, 2010; Crum et al., 2013) provides an organized construct to target and prioritize (in this case, qualities about the process of engaging in health behaviors that are more or less appealing) when crafting public health interventions and thus sheds refreshing light on a novel way to make people more active and eat better.

While the present research intervenes on *individuals’* mindsets, ultimately, our aim is to highlight opportunities for institutions and industries to instill more appealing mindsets that predict healthier behavior. As examples, Studies 2 and 3 demonstrate that mindsets about health can be deliberately improved in real-world contexts, such as through existing health programs, challenging dominant unappealing representations of physical activity and healthy eating that pervade traditional physical and nutrition education.

When implementing this approach, health-behavior practitioners should heed three key clarifications. First, making the overall class or intervention more enjoyable is distinct from making the behavior itself more appealing. While gamified approaches such as guessing the amount of sugar in a food is an engaging class activity, this strategy is limited in that the fun stems from the auxiliary activity (e.g., trivia) and not the health behavior itself (e.g., healthy eating). Second, changing mindsets is not about falsifying ingredients in food or using misleading fitness class descriptions. To the contrary, process mindset theory orients people to appealing qualities that are inseparable to the actual behavior, expanding the repertoire of behaviors that are both healthy and appealing. Third, while mindsets are not inherent to the objective qualities of behaviors, opportunities to improve the experience of health behaviors should not be ignored. For example, improving the preparation of vegetables can provide more taste qualities to highlight and thus help establish more appealing mindsets about healthy eating.

### Limitations and Future Directions

There are a number of limitations of the current studies that warrant future research. First, the samples in Studies 2 and 3 consist of individuals who were already enrolled in fitness and food classes. Although these classes represent real world contexts, this recruitment method may limit our understanding of intervention effects by including participants who already intended to exercise more or improve their diet. With respect to changing mindsets about physical activity or healthy eating, interventions in this study do not exhaust all the ways to alter process mindsets, nor were the studies designed to test avenues by which mindset is changed. For example, Study 2 openly discussed the role of mindset and suggested that participants could learn to develop a more appealing mindset about the process of engaging in exercise. In contrast, food classes in Study 3 did not intervene meta-cognitively on mindset but engaged in a series of experiential exercises that reinforced the pleasure of healthy foods. These and other differences between the two interventions (e.g., study duration) may have contributed to the relatively weaker effects on follow-up behaviors observed in Study 3. This work represents a first proof of principle that changing process mindsets is possible. Future research aimed at understanding how interventions can fully harness mindsets about physical activity and eating healthy to engender sustained changes in health behaviors is warranted.

Experiential learning opportunities (e.g., engaging in physical movements, tasting foods) were likely an important element in our interventions. According to wise intervention theory, opportunities to engage in physical activities and to taste foods provide important leverage points by which to reinforce the mindset. Such experiences provide necessary “soil” in which to develop a “seed,” in this case, the interpretation that exercise and healthy eating are appealing (Walton and Yeager, 2020). When experiential activities and other contextual factors, such as social norms, are lacking or inconsistent with the promoted mindsets, improvements in mindset can be difficult to achieve or maintain (Yeager et al., 2019). This is evident in research on intelligence mindsets and may help explain mixed findings in previous mindset interventions (Walton and Yeager, 2020). Thus, the present research intends to complement, not replace, efforts to increase access to healthy (delicious) foods and safe (fun) recreational spaces, or to make physical activity and healthy eating behavior socially normative.

Lastly, our samples were limited and not representative of the United States population. Compared to the vast number of people who receive some form of health education each year, our samples are small and the majority of participants in each study identified themselves as non-White. As research on process mindset expands, studies should be replicated with larger, more representative samples as well as incorporate food and movement preferences enjoyed by those who differ by race/ethnicity and SES (Oyserman et al., 2007).

### Conclusion

As the list of behaviors people need to engage in to be healthy continues to expand, being healthy fundamentally represents a *process* more than ever before. Perhaps the shortcomings of traditional health-focused approaches is not a lack of public health resources or outreach (Center for Medicare & Medicaid Services, 2018), but a lack of emphasis on the appealing qualities of those behaviors. Notwithstanding the potential benefits to individual and public health, recognizing the appeal of physical activity and nutritious foods is an important next step toward a culture of healthy living that people could actually enjoy.

## Data Availability Statement

The datasets presented in this study can be found in online repositories. The names of the repository/repositories and accession number(s) can be found below: https://osf.io/pvfea/.

## Ethics Statement

The studies involving human participants were reviewed and approved by Stanford University Institutional Review Board. Written informed consent from the participants’ legal guardian/next of kin was not required to participate in this study in accordance with the national legislation and the institutional requirements.

## Author Contributions

DB and AC developed the study concept and drafted the manuscript. DB performed the data analysis and interpretation in collaboration with AC. All authors contributed to study design and supported the data collection, provided revisions and approved the final version of the manuscript for submission.

## Conflict of Interest

The authors declare that the research was conducted in the absence of any commercial or financial relationships that could be construed as a potential conflict of interest.

## Publisher’s Note

All claims expressed in this article are solely those of the authors and do not necessarily represent those of their affiliated organizations, or those of the publisher, the editors and the reviewers. Any product that may be evaluated in this article, or claim that may be made by its manufacturer, is not guaranteed or endorsed by the publisher.

## References

[B1] Ainsworth B. E. Haskell W. L. Whitt M. C. Irwin M. L. Swartz A. M. Strath S. J. (2000). Compendium of physical activities: an update of activity codes and MET intensities. *Med. Sci. Sports Exerc.* 32 S498–S516. 10993420 10.1097/00005768-200009001-00009

[B2] Auster-Gussman L. A. Rothman A. J. (2018). Understanding the prevalence and correlates of implicit theories of weight in the United States: insights from a nationally representative sample. *Psychol. Health* 33 483–498. 10.1080/08870446.2017.1373112 28911240

[B3] Berkman E. T. Hutcherson C. A. Livingston J. L. Kahn L. E. Inzlicht M. (2017). Self-control as value-based choice. *Curr. Dir. Psychol. Sci.* 26 422–428. 10.1177/0963721417704394 29335665 PMC5765996

[B4] Berridge K. C. Robinson T. E. (2003). Parsing reward. *Trends Neurosci.* 26 507–513. 10.1016/S0166-2236(03)00233-9 12948663

[B5] Center for Medicare & Medicaid Services (2018). *National Health Expenditure Data 2017 Highlights.* Available online at: https://www.cms.gov/Research-Statistics-Data-and-Systems/Statistics-Trends-and-Reports/NationalHealthExpendData/Downloads/highlights.pdf (accessed November 4, 2019).

[B6] Craig C. L. Marshall A. L. Sjöström M. Bauman A. E. Booth M. L. Ainsworth B. E. (2003). International physical activity questionnaire: 12-country reliability and validity. *Med. Sci. Sports Exerc.* 35 1381–1395. 12900694 10.1249/01.MSS.0000078924.61453.FB

[B7] Crum A. J. Leibowitz K. A. Verghese A. (2017). Making mindset matter. *BMJ* 356:j674. 10.1136/bmj.j674 28202443

[B8] Crum A. J. Salovey P. Achor S. (2013). Rethinking stress: the role of mindsets in determining the stress response. *J. Pers. Soc. Psychol.* 104 716–733. 10.1037/a0031201 23437923

[B9] Custers R. Aarts H. (2005). Positive affect as implicit motivator: on the nonconscious operation of behavioral goals. *J. Pers. Soc. Psychol.* 89 129–142. 10.1037/0022-3514.89.2.129 16162049

[B10] DellaVigna S. Malmendier U. (2006). Paying not to go to the gym. *Am. Econ. Rev.* 96 694–719. 10.1257/aer.96.3.694

[B11] Duckworth A. L. Gross J. J. (2020). Behavior change. *Organ. Behav. Hum. Decis. Process.* 161 39–49. 33716396 10.1016/j.obhdp.2020.09.002 PMC7946166

[B12] Dweck C. S. (2008). Can personality be changed? The role of beliefs in personality and change. *Curr. Dir. Psychol. Sci.* 17 391–394. 10.1111/j.1467-8721.2008.00612.x

[B13] Dweck C. S. Yeager D. S. (2019). Mindsets: a view from two eras. *Perspect. Psychol. Sci.* 14 481–496. 10.1177/1745691618804166 30707853 PMC6594552

[B14] Godin G. Kok G. (1996). The theory of planned behavior: a review of its applications to health- related behaviors. *Am. J. Health Promot.* 11 87–98. 10.4278/0890-1171-11.2.87 10163601

[B15] Hall K. D. (2013). Diet versus exercise in “The Biggest Loser” weight loss competition. *Obesity* 21 957–959. 10.1002/oby.20065 23404767 PMC3660472

[B16] Jamieson J. P. Crum A. J. Goyer J. P. Marotta M. E. Akinola M. (2018). Optimizing stress responses with reappraisal and mindset interventions: an integrated model. *Anxiety Stress Coping* 31 245–261. 10.1080/10615806.2018.1442615 29471669

[B17] Jerusalem M. Schwarzer R. (2014). “Self-efficacy as a resource factor in stress appraisal processes,” in *Self-Efficacy: Thought Control of Action*, ed. Schwarzer R. (London: Hemisphere Publishing), 195–214.

[B18] Lee P. H. Macfarlane D. J. Lam T. H. Stewart S. M. (2011). Validity of the international physical activity questionnaire short form (IPAQ-SF): a systematic review. *Int. J. Behav. Nutr. Phys. Act.* 8:115. 22018588 10.1186/1479-5868-8-115 PMC3214824

[B19] Levine C. S. Miyamoto Y. Markus H. R. Rigotti A. Boylan J. M. Park J. (2016). Culture and healthy eating: the role of independence and interdependence in the United States and Japan. *Pers. Soc. Psychol. Bull.* 42 1335–1348. 10.1177/0146167216658645 27516421 PMC5023492

[B20] Levy B. R. (2009). Stereotype embodiment: a psychosocial approach to aging. *Curr. Dir. Psychol. Sci.* 18 332–336. 10.1111/j.1467-8721.2009.01662.x 20802838 PMC2927354

[B21] Levy B. R. Myers L. M. (2004). Preventive health behaviors influenced by self-perceptions of aging. *Prev. Med.* 39 625–629. 10.1016/j.ypmed.2004.02.029 15313104

[B22] Lipsey M. W. Wilson D. B. (2001). *Practical Meta-Analysis.* Thousand Oaks, CA: SAGE.

[B23] Luszczynska A. Scholz U. Schwarzer R. (2005). The general self-efficacy scale: multicultural validation studies. *J. Psychol.* 139 439–457. 10.3200/jrlp.139.5.439-457 16285214

[B24] Mann T. Tomiyama A. J. Ward A. (2015). Promoting public health in the context of the “Obesity epidemic”: false starts and promising new directions. *Perspect. Psychol. Sci.* 10 706–710. 10.1177/1745691615586401 26581722 PMC4654677

[B25] Markus H. R. Kitayama S. (2010). Cultures and selves: a cycle of mutual constitution. *Perspect. Psychol. Sci.* 5 420–430. 10.1177/1745691610375557 26162188

[B26] Markus H. R. Zajonc R. B. (1985). “The cognitive perspective in social psychology,” in *Handbook of Social Psychology*, 3rd Edn, Vol. 1 eds Lindzey G. Aronson E. (New York, NY: Random House), 137–230.

[B27] Moore L. V. Thompson F. E. (2015). Adults meeting fruit and vegetable intake recommendations — United States, 2013. *Morb. Mortal. Wkly. Rep.* 64 709–713. PMC4584842 26158351

[B28] Murphy M. C. Dweck C. S. (2010). A culture of genius: how an organization’s lay theory shapes people’s cognition, affect, and behavior. *Pers. Soc. Psychol. Bull.* 36 283–296. 10.1177/0146167209347380 19826076

[B29] O’Dea J. A. (2003). Why do kids eat healthful food? Perceived benefits of and barriers to healthful eating and physical activity among children and adolescents. *J. Am. Diet. Assoc.* 103 497–501. 10.1053/jada.2003.50064 12669014

[B30] Oyserman D. Fryberg S. A. Yoder N. (2007). Identity-based motivation and health. *J. Pers. Soc. Psychol.* 93 1011–1027. 10.1037/0022-3514.93.6.1011 18072851

[B31] Preacher K. J. Hayes A. F. (2004). SPSS and SAS procedures for estimating indirect effects in simple mediation models. *Behav. Res. Methods Instrum. Comput.* 36 717–731. 10.3758/BF03206553 15641418

[B32] Puisais J. (1995). L’éducation du goût. *C. R. Séances Acad. Agric. France* 81 3–16.

[B33] Rozin P. Fischler C. Imada S. Sarubin A. Wrzesniewski A. (1999). Attitudes to food and the role of food in life in the U.S.A., Japan, Flemish Belgium and France: possible implications for the diet-health debate. *Appetite* 33 163–180. 10.1006/appe.1999.0244 10502362

[B34] Ryan R. M. Deci E. L. (2000). Intrinsic and extrinsic motivations: classic definitions and new directions. *Contemp. Educ. Psychol.* 25 54–67. 10.1006/ceps.1999.1020 10620381

[B35] Turnwald B. P. Jurafsky D. Conner A. Crum A. J. (2017). Reading between the menu lines: are restaurants’ descriptions of “healthy” foods unappealing? *Health Psychol.* 36:1034. 10.1037/hea0000501 28541069

[B36] Walton G. M. Yeager D. S. (2020). Seed and soil: psychological affordances in contexts help to explain where wise interventions succeed or fail. *Curr. Dir. Psychol. Sci.* 29 219–236. 10.1177/0963721420904453 32719574 PMC7384695

[B37] Woolley K. Fishbach A. (2016). For the fun of it: harnessing immediate rewards to increase persistence in long-term goals. *J. Consum. Res.* 42 952–966. 10.1097/MD.0000000000000215 25526439 PMC4603125

[B38] Woolley K. Fishbach A. (2018). It’s about time: earlier rewards increase intrinsic motivation. *J. Pers. Soc. Psychol.* 114 877–890. 10.1037/pspa0000116 29771568

[B39] Wyche J. Nicholson L. Lawson E. Allensworth D. (1997). *Schools and Health: Our Nation’s Investment.* Washington, DC: National Academy Press. 25121262

[B40] Yeager D. S. Hanselman P. Walton G. M. Murray J. S. Crosnoe R. Muller C. (2019). A national experiment reveals where a growth mindset improves achievement. *Nature* 573 364–369. 10.1038/s41586-019-1466-y 31391586 PMC6786290

