# Supplementary material for: Can Exercising and Eating Healthy Be Fun and Indulgent Instead of Boring and Depriving? Targeting Mindsets About the Process of Engaging in Healthy Behaviors
Source: Front Psychol. 2021 Oct 5;12:745950. doi: 10.3389/fpsyg.2021.745950 (PMC8546637; doi:10.3389/fpsyg.2021.745950)
Supplement: Supplementary file 1 [file Data_Sheet_1.docx]

Supplementary Material

Table of Contents

Preliminary Studies on Scale Development

Item Generation**2**

Item Testing**4**

Supplementary Analyses for Study 1

Factor Analysis and Reliability**8**

Supplementary Materials for Study 2

Appeal-focused Intervention**10**

Health-focused Intervention**11**

Supplementary Materials for Study 3

Schedule of activities**12**

Intervention Content**12**

Preliminary Studies (Scale Development)

**Item Generation: What are people’s mindsets about the process of being healthy?**

The purpose of this initial study was to generate a range of potential items reflecting people’s mindsets about the process of being healthy.

**Method**

Potential items were generated from qualitative data reported in: Conner, Boles, Markus, Eberhardt, & Crum (2019). Americans’ health mindsets: Content, cultural patterning, and associations with physical and mental health. *Annals of Behavioral Medicine, 53*(4), 321-332. See Conner et al. (2019) for additional details (methods, sample demographics).

Mindsets can vary widely and are not always consciously accessible or easily articulated (hence why they are sometimes referred to as “implicit theories”). Therefore, we began our investigation into the range of potentially important mindsets about what the process of health is like based on existing qualitative research that asked participants a broad but potentially revealing question: “What keeps you from becoming your healthiest self?” Two trained research assistants reviewed participants’ open-ended responses to this question and identified all themes reflecting barriers or potentially unhelpful assumptions about being healthy, such as engaging in health behaviors. Then a team of five subject matter experts on theories of mindset, motivation, and health behavior refined the themes and determined an opponent anchor for each item (e.g., identifying “relaxing” to represent the opposite of “stressful”).

**Results and Discussion**

Discussions among experts led to several reductions and consolidations of the RA-generated list of themes, resulting in 10 themes reflecting a spectrum of potential mindsets about the process of being healthy. These 10 themes and examples of participant responses are depicted in Table S1.

**Table S1.** *Example Responses to “What keeps you from becoming your healthiest self?” and Corresponding Themes Describing Mindsets about the Process of Being Healthy*

| Process Mindset Theme | Example |
| --- | --- |
| Difficult/Easy | "Laziness" "Trying hard to stick to a diet" |
| Painless/Painful | "Arthritis, joint damage" "My mobility in my knees, I'm in pain everyday" |
| Unpleasant/Pleasurable | "Good fast food" |
| Stressful/Relaxing | "Unfortunately a healthy diet… takes effort and sometimes the amount of effort becomes stressful." |
| Time-saving/Time-consuming | "No time to cook or exercise" "Time management" |
| Inconvenient/Convenient | "Not having access to healthy food" |
| Boring/Fun | "Trying to get new ideas for weight loss and exercise that is fun not boring" |
| Cheap/Expensive | "Lack of being able to buy good ingredients" "I can't afford to eat great" |
| Lonely/Social | "Keeping everyone else around me healthy, no time for myself" "Busy with family" |
| Depriving/Indulgent | "Temptation" "Cravings of indulgent foods" |

**Item Testing: Can people’s mindsets about the process of being healthy be reliably measured?**

The purpose of this study was to turn the list of mindset themes generated from analyses of qualitative responses into a set of quantitative scales for measuring mindsets about the process of being healthy and then to examine the factor structure and internal reliability for the scales. To do this, we put each mindset theme on a 4-point response scale, with higher values representing the more appealing qualities (e.g., 1 = *Very boring*, 2 = *Somewhat boring*, 3 = *Somewhat fun*, 4 = *Very fun*). To capture people’s mindsets about the process of being healthy, broadly speaking, as well behavior-specific mindsets about physical activity and healthy eating, we developed three separate scales: one that asked people to reflect on their mindsets regarding the process of health generally (MPH-General), one focused on exercise (MPH-Physical Activity) and a third, focused on healthy eating (MPH-Healthy Eating). Using a new diverse sample, we explored the initial internal consistency and factor structure of each scale.

**Method**

**Participants and Procedure.**  415 online participants recruited via Dynata, formerly known as Survey Sampling International, completed our survey. Demographic characteristics of the sample are presented in Table S2. Age of participants ranged from 25 to 80 years old, with a mean age of 40 years (*SD* = 11.73). First, participants completed the MPH-General according to the instructions: “The following statements are different opinions about what it is like to become healthy or maintain good health. Please select the option in each row that best describes how you feel about the process of becoming healthy and maintaining good health.” Afterwards, they completed two additional behavior-specific scales, MPH-Physical Activity and MPH-Healthy Eating, which instructed participants to “select the option in each row that best describes how you feel about engaging in *physical activity*” and “how you feel about *eating healthy*”, respectively.

**Table S2.** *Sample Demographic Characteristics for Item Testing (Preliminary Study) and Study 1*

| Characteristic | |  |  | Preliminary Study  (Item Testing) | |  | Study 1 | |
| --- | --- | --- | --- | --- | --- | --- | --- | --- |
|  |  |  |  | N | % |  | N | % |
| Gender | |  |  |  |  |  |  |  |
|  | Male |  |  | 202 | 48.7% |  | 275 | 51.3% |
|  | Female |  |  | 213 | 51.3% |  | 261 | 48.7% |
| Ethnicity | |  |  |  |  |  |  |  |
|  | African American | |  | 100 | 24.1% |  | 163 | 30.4% |
|  | Asian American | |  | 103 | 24.8% |  | 121 | 22.6% |
|  | European American | |  | 122 | 29.4% |  | 143 | 26.7% |
|  | Latinx American | |  | 90 | 21.7% |  | 109 | 20.3% |
| Education | |  |  |  |  |  |  |  |
|  | No BA Degree | |  | 251 | 60.5% |  | 277 | 51.7% |
|  | BA Degree | |  | 164 | 39.5% |  | 259 | 48.3% |
| N |  |  |  | 415 |  |  | 536 |  |

**Results and Discussion**

First, each scale item was screened for appropriate item endorsement (i.e., mean) and variability (i.e., standard deviation). All items had moderate means (i.e., between 2 and 3 on the 4-point scale), suggesting no ceiling or floor effects, while standard deviations suggested sufficient variability (all *SDs* > .7) (see Table S3). Prior to conducting an exploratory factor analysis (EFA) of MPH-General, we ran a parallel analysis (Horn, 1965) to obtain a better approximation of how many factors underlie the 10 items. Parallel analyses and EFAs were run using the “fa.parallel” and “fa” functions in R’s *psych* package (version 1.8.12), and employed maximum likelihood extraction with direct oblimin rotation to allow factors to correlate in models that involved more than one factor (Costello & Osborne, 2005).

Parallel analyses suggest that we extract no more than 2 factors in subsequent EFAs (cumulative variance explained = 48%). Ultimately, we sought a single-factor structure in order to create a concise measure for mindsets along a single dimension (e.g., appeal). EFA results revealed three items with low loadings (≤.4) along a single-factor solution, and low inter-item correlations (<.2): Painful/Painless, Time-consuming/Time-saving, and Expensive/Cheap. Based on these statistical interpretations, as well as our theoretical considerations (items were either redundant with other items or they were ambiguous with respect to how they mapped on to the dimension of appeal)^[[1]](#footnote-1)^, we removed these 3 items from our analyses and the parallel analysis and EFA was rerun with the remaining items. Results support a single-factor solution for the 7-item MPH-General (eigenvalue = 3.22, explained variance = 37%; Cronbach’s α = .80; M = 2.59, SD = .52). We also ran factor analyses for these 7 items adapted for MPH-Physical Activity (Cronbach’s α = .86; M = 2.54, SD = .54) and MPH-Healthy Eating (Cronbach’s α = .86; M = 2.50, SD = .53) and found all scales to demonstrate internally consistent single-factor structures (Table S3).

**Table S3.** *Exploratory Factor Analysis Results for MPH-General, MPH-Physical Activity, and MPH-Healthy Eating*

| Item | | | | Factor Loading | | Mean | | SD | |  |
| --- | --- | --- | --- | --- | --- | --- | --- | --- | --- | --- |
| *MPH-General* | |  | |  | |  | |  | |  |
|  | Stressful...Relaxing | | | | 0.76 | | 2.54 | | 0.79 | |
|  | Unpleasant...Pleasurable | | | | 0.67 | | 2.79 | | 0.80 | |
|  | Difficult...Easy | | | | 0.57 | | 2.42 | | 0.79 | |
|  | Depriving...Indulgent | | | | 0.57 | | 2.45 | | 0.77 | |
|  | Inconvenient...Convenient | | | | 0.57 | | 2.48 | | 0.78 | |
|  | Boring...Fun | | | | 0.56 | | 2.72 | | 0.74 | |
|  | Lonely...Social | | | | 0.53 | | 2.72 | | 0.74 | |
|  |  | |  | |  | |  | |  | |
| *MPH-Eating Healthy* | | | |  | |  | |  | |  |
|  | Unpleasant...Pleasurable | | | | 0.78 | | 2.73 | | 0.80 | |
|  | Boring...Fun | | | | 0.74 | | 2.60 | | 0.80 | |
|  | Stressful...Relaxing | | | | 0.74 | | 2.70 | | 0.84 | |
|  | Difficult...Easy | | | | 0.71 | | 2.47 | | 0.85 | |
|  | Depriving...Indulgent | | | | 0.69 | | 2.37 | | 0.77 | |
|  | Inconvenient...Convenient | | | | 0.62 | | 2.45 | | 0.82 | |
|  | Lonely...Social | | | | 0.57 | | 2.63 | | 0.77 | |
|  |  | |  | |  | |  | |  | |
| *MPH-Physical Activity* | | | |  | |  | |  | |  |
|  | Unpleasant...Pleasurable | | | | 0.78 | | 2.62 | | 0.86 | |
|  | Stressful...Relaxing | | | | 0.77 | | 2.72 | | 0.84 | |
|  | Boring...Fun | | | | 0.70 | | 2.79 | | 0.86 | |
|  | Difficult...Easy | | | | 0.70 | | 2.43 | | 0.89 | |
|  | Depriving...Indulgent | | | | 0.64 | | 2.61 | | 0.80 | |
|  | Inconvenient...Convenient | | | | 0.60 | | 2.46 | | 0.85 | |
|  | Lonely...Social | | | | 0.55 | | 2.75 | | 0.77 | |
| *Note*. N=415 | |  | |  | |  | |  | |  |

**Supplementary Analyses for Study 1**

**Factor Structure.** Results from confirmatory factor analyses (factor loading and item descriptive statistics) are shown in Table S4. Model fit statistics confirmed that a one-factor model had good fit for MPH-Physical Activity (X^2^(14) = 45.492, CFI = .984, TLI = .976, RMSEA = .065, 90% CI[.044, .086]) and MPH-Healthy Eating (X^2^(14) = 42.209, CFI = .985, TLI = .978, RMSEA = .061, 90% CI[.041, .083]). Each scale demonstrated appropriate standardized factor loadings (MPH-Physical Activity α’s = .63 - .85; MPH-Healthy Eating α’s = .60 - .82).

**Table S4.** *Confirmatory Factor Analysis* *for MPH-Physical Activity and MPH-Healthy Eating*

| Scale Item | | Standardized Factor Loadings | Mean | SD |
| --- | --- | --- | --- | --- |
| MPH-Physical Activity | |  |  |  |
|  | Unpleasant...Pleasurable | 0.85 | 2.67 | 0.84 |
|  | Stressful...Relaxing | 0.79 | 2.80 | 0.85 |
|  | Boring...Fun | 0.79 | 2.65 | 0.88 |
|  | Difficult...Easy | 0.75 | 2.43 | 0.88 |
|  | Inconvenient...Convenient | 0.73 | 2.46 | 0.80 |
|  | Indulgent...Depriving | 0.68 | 2.58 | 0.78 |
|  | Lonely...Social | 0.63 | 2.73 | 0.76 |
| MPH-Healthy Eating | |  |  |  |
|  | Boring...Fun | 0.82 | 2.59 | 0.80 |
|  | Unpleasant...Pleasant | 0.81 | 2.74 | 0.83 |
|  | Stressful...Relaxing | 0.79 | 2.71 | 0.78 |
|  | Depriving...Indulgent | 0.75 | 2.36 | 0.76 |
|  | Difficult...Easy | 0.74 | 2.51 | 0.82 |
|  | Lonely...Social | 0.71 | 2.64 | 0.70 |
|  | Inconvenient...Convenient | 0.60 | 2.49 | 0.79 |
| *Note.* N=536. Estimates were calculated for Time 1 outcomes. Items for each scale are in descending order of factor loading | | | | |
|  |  |  |  |  |

**Internal Consistency and Test-Retest Reliability.** The 7-item MPH-Physical Activity and MPH-Healthy Eating had moderate means, good variability, and generally good internal consistency over time (**Table S5**). In this sample, alphas at Time 2 (3 months) were slightly lower than Time 1 scores on MPH-Physical Activity and MPH-Healthy Eating, but still showed acceptable fit. Zero-order correlations among the three time points reveal good test-retest stability.

**Table S5.** *Descriptive Statistics and Test-Retest Reliability of MPH-Physical Activity and MPH-Healthy Eating over 3 Months*

|  |  |  |  |  | Zero-order Correlations | |
| --- | --- | --- | --- | --- | --- | --- |
| Variable | M | SD | Cronbach's α |  | T1 | T2 |
| MPH-Physical Activity (*n* = 285) | | | | | | |
| T1 | 2.63 | .69 | .91 |  | - | - |
| T2 | 2.58 | .51 | .67 |  | .76 | - |
| MPH-Healthy Eating (*n* = 285) | | | | | | |
| T1 | 2.57 | .65 | .92 |  | - | - |
| T2 | 2.54 | .47 | .66 |  | .76 | - |
| *Note.* All zero-order correlations are significant at *p* < .001.  Time 1 and Time 2 were separated by 3 months. | | | | | | |
|  |  |  |  |  |  |  |

**Supplementary Materials for Study 2**

**Appeal-Focused Intervention**

*Intervention Script*

Hello everyone, I hope you’re having a good day and enjoying your [type of exercise] class so far. Today, I wanted to take a few minutes to speak with you all about the impact our mindsets can have on the way we view exercise, stick with fitness-related goals, and even impact the health benefits we receive from exercise. In psychology, we think of mindsets as the core associations individuals hold about the nature or processes of things in the world around them. For example, someone may have the mindset that the process of eating healthy is that it’s disgusting and depriving while someone else may hold the mindset that eating healthy food is a relaxing or indulgent process. You can imagine how such mindsets can shape one’s subjective experience while performing these behaviors.

I want to start by asking you all what are some associations that come to mind for you right now when you think about exercise? In other words, fill in the blank about what the process of exercising is like: Exercising is _____ (await participants’ answers).

These statements, associations between exercise and qualities like 'relaxing’ or ‘fun’ are a window into our mindsets about physical activity. However, when it comes to exercise, many may view the process of physical activity and exercise as work or a time-consuming activity that we don’t have time for. Others may have the mindset of “no pain, no gain” – that exercise is something unappealing we have to endure. And some even consider exercise as an embarrassing activity that leaves us in discomfort. Yet, preliminary research shows that these *unappealing* mindsets about the process of exercising correlate with lower engagement in physical activity. On the flip side, there are ways to change our mindset about exercising to be one that recognizes it as a rewarding process. If you view exercise as something that is indulgent, relaxing, convenient, or pleasurable, you’ll probably be more likely to engage it in.

What are some reasons we can think of for how exercise *is* fun, indulgent, easy, relaxing, convenient, pleasurable? (allow 1 min)

It’s great you’re all in a group class, but what are some other ways we can make daily exercise more social? (allow 1min) Great answers. [Insert personal anecdote, e.g. ‘I was talking to my friend about the potential role of mindsets in exercise since one of his New Years’ resolutions is to get back in shape. Now he’s always asking me to go to the gym together, particularly for the exercises he hates the most, he’s noticed that he’s more likely to work out when he goes with a friend. And now I get the added benefit of better abs, too.]

I wanted to share this information with you today to just get you thinking about the mindsets and how they’re related to exercise. If you take a look around the room, there is a group of students in here ready to exercise by doing a fun activity. Maybe you’ll meet someone new by getting exercise in this class; maybe this class will help you relax from the stress of the day. But think about it – you’re not stressed in an academic class right now having to worry about memorizing or regurgitating information. You’re getting to connect with people in a unique way.

Realizing exercise as an appealing process helps sustain our motivation and I hope this class inspires you to discover all the joys of movement. Look fondly at exercise as a fun and social activity, do the things that make you feel like “once you start, you just can’t stop”. Thank you again for your time, and enjoy the rest of your quarter in class. I will be returning to this class in Week 10 to deliver a post-survey for you to fill out.

**Health-Focused Intervention**

*Intervention Script*

Hello everyone, I hope you’re enjoying your day so far. Today, I just wanted to take a few minutes to emphasize to you all the importance of exercising and the benefits of physical activity for your health and well-being.

But I want to see what you know about it first. Can anyone tell me some health benefits of exercising? (allow 1 min for this)

According to the Center for Disease Control, or the CDC, there are a number of health benefits related to getting a regular amount of exercise. First off, regular physical activity can help you maintain your weight, and if you combine physical activity with a healthy diet, you are more likely to lose weight. The CDC’s website has some great tools and information to get you started, where you can learn about even more health benefits than maintaining or losing weight. A maintained regimen of daily exercise lowers your risk of cardiovascular disease, high blood pressure, and the likelihood of acquiring Type 2 diabetes. And the more physical exercise you do, the lower the risk becomes. Moderate-intensity aerobic exercise can also reduce the risk of certain types of cancer, such as lung and breast cancer, compared to people who are not active. And in so doing, regular physical activity increases your chances of living longer, since you are less at risk of dying early from leading causes of death like heart disease. Other health benefits of regular physical activity are stronger muscles and bones, a fortification of the immune system, and an increase in your energy levels. Maybe one of the greatest benefits of regular physical activity is that it also improves your mental health by lowering your risk of depression and helping you sleep better. The best part is that virtually anyone can gain these benefits from regular physical activity, no matter your age, ethnicity, shape, or size. What matters is your persistence in being active.

Who here knows what the weekly-recommended amount of physical activity is? (await participants’ answers) According to the CDC, it is recommended that everyone participate in moderate-intensity physical activity for at least 150 minutes, or 2 hours and 30 minutes, per week, basically 30 minutes of exercise on each of five days. This can be activities such as walking at a brisk pace or swimming. But for those who are capable, the CDC alternatively recommends at least 75 minutes of vigorous-intensity aerobic activity, like running or cycling. Or you can mix moderate and vigorous activity to your liking. By getting at least two and a half hours of moderate physical activity, you can get all of those health benefits that I mentioned earlier.

By taking this [type of exercise] class, you are already on your way to getting your recommended weekly amount of physical activity. Spending two hours per week playing [type of exercise], all of these health benefits are on their way to contributing to your well-being. Thank you for your time. I will be returning to your class at the last session to deliver a post-survey for you to fill out.

**Supplementary Materials for Study 3**

**Schedule of Activities**

**Table S6.** *Schedule of Activities for All Classes*

| **Time** | **Activity** |
| --- | --- |
| 0:00 – 0:05 | Assent & Pre-intervention Survey |
| 0:05 – 0:20 | Lesson Plan |
| 0:20 – 0:30 | Food Sampling |
| 0:30 – 0:40 | Recipe Demonstration |
| 0:40 – 0:45 | Post-intervention Survey |

Note: Schedule above represents activities of all classes across both conditions.

**Table S7.** *Intervention Content for Appeal-Focused versus Health-Focused Conditions*

| **Activity** | **Appeal-focused Condition** | **Health-focused Condition** |
| --- | --- | --- |
| Lesson Plan | - Discussed favorite fruits and vegetables  - Appreciated diversity of fruits and vegetables  - Shared social and seasonal activities with food | - Reviewed official dietary recommendations  - Learned to read nutrition label information  - Discussed food tracking tools and strategies |
| Food Sampling | - Emphasized taste, texture, and sensory appeal of fruits and vegetables | - Emphasized nutrient content and health benefits of fruits and vegetables |
| Recipe Demonstration | - Shared ingredients for a "Summer Sipper" | - Shared ingredients for a "Healthy Green Smoothie" |

1. *Painful/painless* was removed because it did not reflect the full spectrum of the unappealing to appealing process (the absence of pain is not necessarily appealing). *Cheap/expensive* was removed because being cheap is not necessarily appealing to some people and research suggests that being expensive can be appealing because it can convey greater quality (Plassmann, O’Doherty, Shiv, & Rangel, 2008). We removed *time-consuming/time-saving* because its relationship to unappealing/appealing was also ambiguous (exercise that is time-consuming is not necessarily unappealing if the time spent was fun). [↑](#footnote-ref-1)
